# The Multiple Sclerosis Modulatory Potential of Natural Multi-Targeting Antioxidants

**DOI:** 10.3390/molecules27238402

**Published:** 2022-12-01

**Authors:** Panagiotis Theodosis-Nobelos, Eleni A. Rekka

**Affiliations:** 1 Department of Pharmacy, School of Health Sciences, Frederick University, Nicosia 1036, Cyprus; 2 Department of Pharmaceutical Chemistry, School of Pharmacy, Aristotle University of Thessaloniki, 54124 Thessaloniki, Greece

**Keywords:** multiple sclerosis, oxidative stress, neurodegeneration, natural antioxidants, antioxidant bioactive compounds, inflammation

## Abstract

Multiple sclerosis (MS) is a complex neurodegenerative disease. Although its pathogenesis is rather vague in some aspects, it is well known to be an inflammatory process characterized by inflammatory cytokine release and oxidative burden, resulting in demyelination and reduced remyelination and axonal survival together with microglial activation. Antioxidant compounds are gaining interest towards the manipulation of MS, since they offer, in most of the cases, many benefits, due to their pleiotropical activity, that mainly derives from the oxidative stress decrease. This review analyzes research articles, of the last decade, which describe biological in vitro, in vivo and clinical evaluation of various categories of the most therapeutically applied natural antioxidant compounds, and some of their derivatives, with anti-MS activity. It also summarizes some of the main characteristics of MS and the role the reactive oxygen and nitrogen species may have in its progression, as well as their relation with the other mechanistic aspects of the disease, in order for the multi-targeting potential of those antioxidants to be defined and the source of origination of such activity explained. Antioxidant compounds with specific characteristics are expected to affect positively some aspects of the disease, and their potential may render them as effective candidates for neurological impairment reduction in combination with the MS treatment regimen. However, more studies are needed in order such antioxidants to be established as recommended treatment to MS patients.

## 1. Introduction

Multiple sclerosis (MS) is a complex neurodegenerative disease, starting from the early adulthood and up to forty years [1], and concerns chronic peripheral and central nervous system (CNS) inflammation, oxidative stress (OS), as well as blood–brain barrier disruption, followed by neuronal and axonal damage and demyelination [2]. The pathogenesis of MS is rather vague in some aspects; it is well known to be an inflammatory process characterized by the progressive loss of oligodendrocytes, resulting in demyelination and reduced remyelination and axonal survival, whilst microglial activation and antigen presenting cells (APCs) can modulate the immune responses leading to the propagation of the autoimmune MS disease [3]. MS is characterized by relapse and remission clinical phases, with gradual deterioration, during the progression of the disease, with four defined clinical courses, the relapsing-remitting (RR), the primary progressive (PP), the secondary progressive (SP) and progressive-relapsing (PR). Current multiple sclerosis phenotypic classifications include RRMS, clinically isolated syndrome (CIS), radiologically isolated syndrome (RIS), PPMS and SPMS [4]. The RRMS is the main type of manifestation of the disease and is characterized by inflammatory cytokines and oxidative burden, during the relapses, which may, or may not, recover during the remission phase, and may evolve to progressive MS as the neurological deficits and especially axonal damage incline [5].

The aim of this study is to provide information about various currently used, almost on a daily basis, antioxidant compounds, and their derivatives, accentuating their potential role as therapeutic elements or adjuvants in multiple sclerosis. It presents the work that has been performed, mainly in the last decade, on natural antioxidant treatments, with potential pluripotent anti-inflammatory, remyelinating neuroprotective activity, in MS progression, explaining the close interrelation of these conditions in the disease. It also describes examples of how these compounds could be applied as leading molecules for the design of novel derivatives that will directly or indirectly exhibit oxidative stress and MS modulatory effects.

## 2. Inflammation and Oxidative Stress in MS

The etiology of MS remains unknown and most probably consists of a combination of genetic, autoimmunological and environmental factors affecting disturbed immune response; while it includes BBB disturbances, due to alterations of the immune system, resulting in infiltration of T cells into CNS and release of pro-inflammatory cytokines, promoting further the activation of macrophages, and the inflammation of the white matter, causing myelin sheath destruction [6]. The CD4+ T-helper (Th) cells, and especially Th1 and Th17, signal the production of autoantibodies, via the induction of B lymphocytes, decreasing the ratio between T and B lymphocytes, with CD4+, CD8+ and natural killer cells up-regulating the immune responses, whilst regulatory T cells (T-regs), inhibiting the T and B cells, derived autoimmune responses [7]. Various types of leucocytes may increase BBB permeability by stimulating various peripheral cells, such as platelets [8]. Myelin reactive CD4+ T cells perform a key role in demyelination, after their migration into the CNS, whilst naive T lymphocytes maturation to Th1 and Th17 are another myelin specific deterioration mechanism, secreting pro-inflammatory cytokines, with antigen presentation, via major histocompatibility complex II, on dendritic cells, being the main T-cell activation mechanism [9]. B-lymphocytes could also contribute to MS pathogenesis by producing anti-myelin basic protein antibodies [10].

Oxidative stress (OS) is involved in all stages of MS, resulting in lesions on the white and grey matter, being one of the main causes of CNS and peripheral inflammation, and leading to tissue damage [11]. During this damage, cellular immune responses take place, such as immune cells recruitment and proliferation, including phagocytosis and pro-inflammatory cytokines release. They in turn give rise to reactive oxygen (ROS) and nitrogen (RNS) species, which lead to oligodendrocyte and axonal damage, promote the M1 phenotype of macrophages and enhance phagocytic activity [12,13]. ROS and RNS may affect vital components (proteins, lipids, nucleic acids and sugars) and thus lead to cellular distress, apoptosis induction and deregulation of factors such as NF-kB [14]. Neurodegeneration is exacerbated, with the main cells of production being astrocytes, macrophages and microglia, accentuating the interrelation between the inflammatory and oxidant conditions [15]. NO production by microglial cells and astrocytes can be multiplied. Augmented NO, in combination with radicals, forms the oxidant peroxynitrite and leads to neuronal damage and MS progression, also impairing the function of the mitochondrial respiratory chain, leading to electron leakage and further propagation of oxidative injury [16]. Mitochondrial integrity deterioration and electron leakage may lead to decreased ATP synthesis and sodium/potassium pump function in axons, thus enhanced neuronal dysfunction [17]. This dysfunction is further expanded, due to high energy demand of the demyelinating axons and low energy production by mitochondria, influencing the cellular communication and processes of neurons, even before cellular loss occurs [18]. Another crucial participant of oxidative stress is NADPH oxidase-2 (NOX2), producing high amounts of ROS and leading to neuroinflammation in MS, even in low amounts [19]. High amounts of ROS, in activated microglia and macrophages, may result in RNS generation, by the inducible nitric oxide synthase (iNOS) derived nitrogen monoxide, insulting the axonal myelin and the peripheral nervous system, leading to RNS-dependent deterioration of mitochondrial function [20]. These results are in accordance with the data that accumulation of pro-inflammatory stimuli may statistically lead to manifestation of MS, by inflammatory reactions induction, and perhaps the decrease in ROS and RNS levels may lead to their reduction [21]. Mitochondrial dysfunction seems to be a common factor of deterioration seen in many neurodegenerative diseases and among the other factors, aging and mutations in genes expressing antioxidant enzymes seen to be responsible for this [22]. Thus, since OS is a vital cause of brain damage, dietary antioxidants or diet enriched with natural antioxidant supplements may be of assistance towards the confrontation of oxidative stress caused from autoimmune diseases or external stimuli such as heavy metals, pesticides and atmospheric pollutants that may hold responsibility for the imbalance in redox status of the organism [23].

Oxidative modification of lipids results in destruction of lipid rich areas, cellular outer and inner membranes and myelin sheaths, and leads to the production of reactive electron-accepting aldehydes that can modulate the structure of proteins, DNA and RNA, via Schiff base formation [24]. Malondialdehyde (MDA), acrolein, 4-hydroxy-2-nonenal (HNE) and 4-hydroxy-2-hexenal (4-HHE) are end products of the oxidation of polyunsaturated, allylic hydrogen-containing fatty acids which are the most susceptible to oxidation. MDA serves as a marker of lipid peroxidation (LP), together with the evaluation of other markers of oxidative stress, and its levels are significantly high in patients with no treatment, in comparison to those receiving disease modifying therapies, such as interferon-β (INF-β) [25]. These levels are correlated with expanded disability status scale (EDSS), and significant elevation of MDA levels was observed in the blood of RRMS patients, before methylprednisolone administration, and especially during the relapse phase, strengthening the clinical evidence of increased oxidative stress in MS [26]. Furthermore, the mitochondrial ROS (mtROS) production has shown to give rise to a chain of events including NF-kB stimulation and pro-inflammatory cytokine and chemokine induction in endothelial cells, whilst mitochondria targeted antioxidant molecules may at least partly provide anti-inflammatory effects, suppressing NF-kB dependent gene expression, contributing to protection against demyelination and vascular aging [27].

Although oxidative and mitochondrial damages play important role in all degenerative conditions of the CNS, in the progressive stage of MS, the pathways of tissue damage (by comparing the gene expression in active lesions) are more profound [28]. Since MS is a multifactorial disease of the central nervous system (CNS), the etiology of the disease is still not fully understood. Therefore, finding new etiological factors is of such crucial importance. In the acute phase oxidative stress initiates inflammatory processes and in the chronic phase it sustains neurodegeneration. Redox processes in MS are associated with mitochondrial dysfunction, dysregulation of axonal bioenergetics, iron accumulation in the brain, impaired oxidant/antioxidant balance, and oxidative stress memory. Neuronal capability to cope with OS is limited, since low expression of antioxidant defense regulators, such as nuclear factor-erythroid factor 2-related factor 2(Nrf2) and peroxisome proliferator-activated receptor-gamma co-activator (PGC)-1a, seems to take place, especially during the progressive MS, whereas gene deletions in mitochondria may associate with energy deficiency, calcium ions imbalance, axonal degeneration and apoptosis of neurons and oligodendrocytes, that due to the high iron storage may be enhanced together with the radical formation via the Fenton reaction [29]. The liberation of iron, from the myelin sheets during demyelination, may promote the oxidative and mitochondrial injury, involved in plaque formation in white and grey matter [30]. Chronic subclinical extravasation of hemoglobin, in combination with multiple other factors including, but not limited to, dysfunction of different cellular protective mechanisms against extracellular hemoglobin reactivity and oxidative stress, may also be reasons for amplification of the neurodegeneration in MS [31].

## 3. Natural Phenolic Antioxidants

Flanonoids comprise an important part of the phenolic antioxidants. They are colored polyphenols, categorized into six categories: flavones, flavonols, isoflavones, anthocyanins, flavanones and chalcones [32,33]. Their properties are based on conjugation between the A and B rings, and the allocation of hydroxyl, methyl and glycosidic side groups. They possess radical scavenging, metal chelating, antioxidant enzymes promoting, and pro-oxidant enzymes silencing activity, as well as cyclo-oxygenase (COX) and lipoxygenase (LOX) inhibitory effects that lead to anti-inflammatory potential [33,34]. Furthermore, flavonoids can reduce the expression of pro-inflammatory mediators, IL-1β, IL-6, TNF-a, NF-kB, iNOS and MMP-9, and reduce the levels of kinases such as c-jun N-terminal kinase (p-JNK) and MAPK [35].

### 3.1. Quercetin

**Figure 1:**
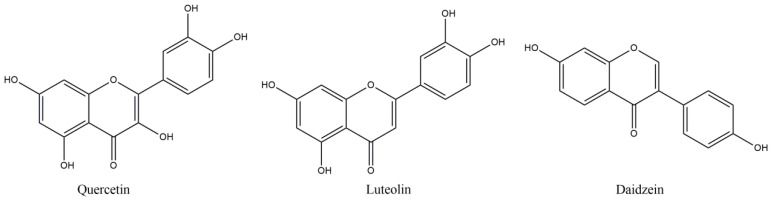
Structures of referred flavonoids.

Among the flavonoids, quercetin (QUE) (Figure 1) holds an important place, with neuroprotective, ROS scavenging, metal chelating and calcium inhibitory effects, but also indirect antioxidant activities, such as the inhibition of enzymes including xanthine oxidase and nitric oxide synthase (NOX) [36]. QUE intraperitoneal administration, in experimental autoimmune encephalomyelitis (EAE) induced mice (EAE is an animal model of MS, produced by administering a myelin basic protein peptide fragment that induces an autoimmune response directed to the myelin sheath surrounding motor neurons), reduced the mean maximum clinical severity up to 2.5 points, in comparison to the control group, whilst it also decreased demyelination more than 50%, as well as inflammation [37]. Furthermore, in vitro treatment of activated T-cells with quercetin in EAE amelioration may be related with IL-12 production inhibition and the blockage of tyrosine phosphorylation of Janus kinase 2 (JAK2), tyrosine kinase 2 (TYK2) and signal transducer and activator of transcription 3 (STAT3), resulting in inhibition of T cell proliferation and Th1 cells differentiation, with parallel INF-γ production decrease reaching up to 87.5% [27]. The immune modulatory effects of QUE may also be related to reduction in TNF-α and IL-1β, as shown from treatment of peripheral blood mononuclear cells, from RRMS patients, with quercetin [38]. In this report, co-treatment of QUE with IFN-β reduced more significantly than the QUE alone, the levels of TNF-α, while matrix metalloproteinase-9 (MMP-9) inhibition consists of another positive asset.

### 3.2. Isoflavones

Phytoestrogens have been reported to show protective effects in EAE, and this effect may partially be linked to various processes that take place in the periphery, including gut function, and in which they seem to implicate [39]. The estrogenic potency concerns QUE and especially isoflavones, such as daidzein. The latter, together with suppressed NF-kB transcriptional activity and inhibition of NO and IL-6 production, may be a therapeutic antioxidant tool for diseases needing multi-targeting treatment [40]. Daidzein oral administration (300 mg/kg/day by oral gavage) in EAE mice led to significant decrease in IFN-γ and IL-12 in splenocytes, whilst IFN-γ was also reduced in the brain, whereas IL-10 secretion was induced in both areas, accentuating the reduction in inflammatory and the induction of anti-inflammatory processes that contributed to the disease severity decrease [41]. Similarly encouraging effects on EAE-induced female Wistar rats were deduced after luteolin (LUT) treatment (10 mg/kg/day i.p.), a flavonoid structurally similar to QUE (Figure 1) [42]. LUT produced up-regulated levels of ciliary neurotropic factor (CNTF), cyclic AMP and total antioxidant capacity with significant decrease in the apoptotic caspase-3, NF-kB and macrophage inflammatory protein-1 alpha (MIP-1α) [42]. These results also showed the substantial decrease by almost three points in the mean values of clinical paralytic score results, reported weekly, with bigger escalation between the treated and non-treated groups as the weeks proceeded. LUT may also be used as a co-treatment, co-administered with palmitoylethanolamide (PEA, an endogenous ethanolamide in CNS with regenerative and protective effect towards the damaged neurons) [43]. Daily intraperitoneal administration of PEA-LUT (composite of ultramicronized palmitoylethanolamide combined with the flavonoid luteolin) improved clinical scores, in a dose-dependent manner. Thus, it caused statistical decreases in the mRNA expression of TNF-α, IL-1β and ΙFN-γ, using the highest dose of 5 mg/kg i.p. The mRNA levels of Toll-like receptor 2 (TLR2), N-formyl peptide receptor 2 (Fpr2), tumor necrosis factor receptor superfamily member 9 (CD137), T cell co-receptor CD3 γ chain (CD3-γ), T cell surface glycoprotein ζ chain (TCR-ζ chain) and cannabinoid receptor type 2 (CB sub-index 2) were significantly reduced in the brainstream and the cerebellum, on the 14th day of the treatment [43].

### 3.3. Epigallocatechin Gallate

Epigallocatechin gallate (EGCG) is the most abundant active catechin, found mainly in green tea. It has shown to decrease MS severity, administered after the appearance of EAE clinical signs, via brain inflammation and demyelination decrease, followed by diminution of the expression of inflammatory cytokines and chemokines and T cell responses [44]. Especially, IL-17 and INF-γ production was related to STAT pathway and retinoid related orphan receptor gamma effects, inhibiting both Th1 and Th17 cell differentiation. These effects on Th1 and Th17 T cells may relate to decrease in IL-6, IL-1β and TNF-a and increase in Treg cells in lymph nodes, spleen and CNS, and inhibition of T-box and retinoid orphan receptor-γt expression (these results came after administration with a diet of 0.15–0.30% EGCG (*w/w*) for 30 days) [45]. Modulation of the plasma levels of intercellular adhesion molecule 1 (ICAM-1) and C-C Motif Chemokine Receptor 6 (CCR6) in CD4 (+) cells was also observed, inhibiting immune infiltration and attenuating clinical signs and delayed type hypersensitivity skin response [45]. The EGCG effects may also partly be derived by the down-regulation of the CD80 and CD86 molecules, resulting in a decrease in T cell proliferation and antigen presentation [46]. EGCG has also shown combinatorial effects against glutamate and TRAIL-induced neuronal cell death, offering regeneration of hippocampal axons in vitro. These effects were also translated in in vivo model, by reducing the clinical severity and the inflammatory infiltration [47]. EGCG has also been shown to increase the gene expression of myelin proteolipid protein (PLP) and oligodendrocyte transcription factor 1 (Olig1) in cuprizone induced demyelination in mice, after four weeks of treatment at 50 mg/kg body weight daily i.p., improving the maintenance of myelin and the myelinogenesis, since PLP and, its alternative spliced isoform, DM20, constitutes more than 50% of the total protein of myelin in the CNS [48].

EGCG has been used as a co-treatment with glatiramer acetate (GA), in stable MS patients, with EDSS scores lower than 4.5 [49]. Since EGCG is known to improve energy metabolism at rest or during exercise, its effects on energy metabolism and substrate utilization was tested (via fasting and postprandial energy expenditure, and fat and carbohydrate oxidation) in these patients, after 600 mg/day per os administration for twelve weeks. There was a better effect in male patients, with postprandial expenditure and carbohydrate oxidation, as well as glucose supply and adipose tissue perfusion being significantly lower in men, and with increased working efficiency fueled mainly by the more stable carbohydrate and not fat oxidation, showing an improved muscle metabolism without any significant changes in systematic lipid, insulin or glucose responses or reported adverse effects [49]. The synergistic effects of GA and EGCG led also to protection from glutamate and tumor necrosis factor-related apoptosis-inducing ligand neuronal death [47].

### 3.4. Arbutin

Arbutin (ARB), a glycosylated hydroquinone (hydroquinone-β-D glucopyranoside), has antioxidant and anti-inflammatory properties, improving memory dysfunction and down-regulating on gene expression of TNF-alpha, IL-6 and glial fibrillary acidic protein, in a dose-dependent manner, resulting in neuronal damage and astrocytes activation attenuation [50]. These effects are accompanied by ROS reducing potential and iNOS activity, together with improved Nrf2 and heme oxygenase-1 (HO-1) levels in various neurological disorders, such as epilepsy, Parkinson’s disease and MS [50]. In the latter case, lysolecithin induced demyelination of the optic chiasm was chosen (micro-injection of 2 μL LPC (1%) into the rat optic chiasm and treatment with ARB with 50 mg/kg, i.p. daily for 2 weeks) [51], since optic nerve demyelination is frequent in MS patients and offers a reliable model of study. ARB improved visual evoked potential (VEP) waves, decreasing P1 latency and improving functional recovery, with substantial reduction in demyelination and enhancement of myelin repair, as it is seen by myelin basic protein (MBP) and oligodendrocyte transcription factor 2 (Olig2), increased expression and enhanced myelin intensity. These functional effects may also be derived by the Nrf2 and HO-1 increased and iNOS decreased mRNA expression levels, as well as by the modulation of the transcription of pro-inflammatory cytokines (IL-1β, IL-17 and TNF-α) and the increase in the anti-inflammatory ones, such as IL-10, that may led to attenuation of glial activation [51].

### 3.5. Arctigenin

**Figure 2:**
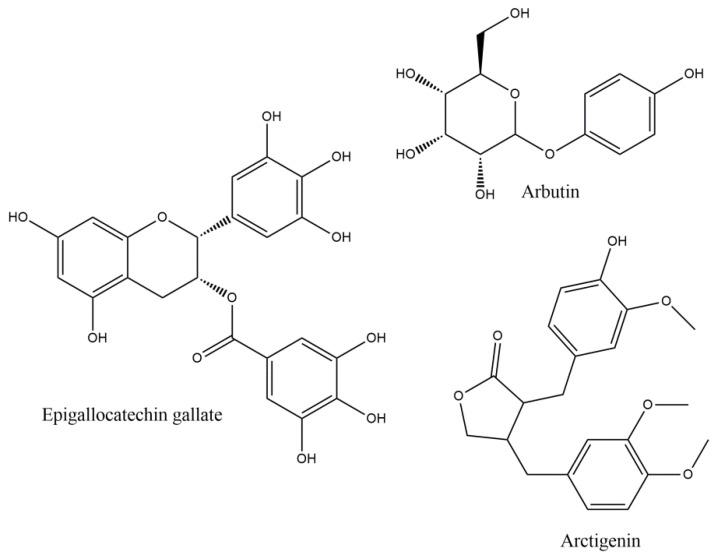
Epigallocatechin gallate, Arbutin and Arctigenin.

Arctigenin (ARC) (Figure 2), a phenylpropanoid dibenzylbutyrolactone lignan, exhibits a variety of pharmacological activities, including the antioxidant, since chronic administration (15 mg/kg/day i.p. for 6 weeks) enhances endurance, increasing the expression of antioxidant related genes, such as superoxide dismutase (SOD), glutathione reductase (GSR), glutathione peroxidase-1 (GPX1) and thioredoxin (Txn), via AMPK (AMP-activated protein kinase) phosphorylation and p53 activation, resulting also in Nrf2 activation [52]. ARC also offers neuroprotection, decreasing neuronal death and inflammatory and oxidative processes that result from microglial activation [53]. PPAR-γ (peroxisome proliferator-activated receptor γ) is a negative regulator of retinoid-related orphan receptor-γt (ROR-γt) that is implicated in Th17 cell differentiation, whilst phosphorylated p38 MAPK (mitogen-activated protein kinase) member could reduce the immune regulating activity of PPAR-γ, by modulating its expression. It has been shown that ARC (in 10 mg/kg) decreases the demyelination and inflammation in EAE model by peripheral inhibition of Th1 and Th17 cells, with substantial reduction in gene expression of ROR-γt, IL-17A and IFN-γ, with, interestingly, restraining only of Th17 cells differentiation in the brain, by AMPK activation and p53 phosphorylation, which leads to PPAR-γ up-regulation [54].

### 3.6. Oleuropein

**Figure 3:**
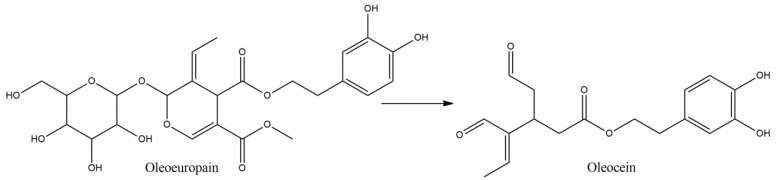
Oleuropein and its secoiridoid derivative, oleacein.

Oleuropein (OL) is a hydroxytyrosol derivative, comprising also an elanolic acid and glucose unit, is found in olive trees and is present in greater amounts in its leaves and unprocessed fruits that under the β-glucosidase activity can be transformed to its de-glycosylated form, the demethyloleuropein and oleuropein aglycone (OLA) [55]. The bioavailability of OL seems satisfactory, with 50–60% intestinal absorption, involving a glucose transporter-mediated carrier [55]. The lipophilic aglycone compounds of OL succeed in BBB crossing by passive diffusion, after liver metabolism [56]. OL has been found to exert neuroprotective, antioxidant and anti-inflammatory effects, by various mechanisms, against many stimuli inducing neurotoxicity and memory deficits, affecting antioxidant capacity and memory impairment, with LP and apoptosis regulation, by modulating SOD, GPx and Bcl2/Bax proteins [57]. As far as MS is concerned, olive leaf extract containing 45.96 mg/kg of OL, was administered the eighth day after the induction of EAE, and tested for ten successive days [58]. The intensity of clinical scores was attenuated, at the peak of the disease, about 67%, affecting also the relapse time and score and the death rate that was declined from 88% to 13%. The GPx and TBARs level were significantly increased and decreased, respectively, whilst the SOD activity increased compared to untreated animals, but not significantly. Furthermore, sirtuin-1 (SIRT1) protein expression levels were found increased in brain tissue, including microglial cells, during both relapse and remission phases, especially in dentate gyrus and subventricular zone, with up-regulation of the anti-inflammatory M2 type and down-regulation of proinflammatory M1 type microglia, and myelin integrity preservation. A secoiridoid derivative of OL, is oleacein (OLE) (Figure 3), a dialdehyde of decarboxymethyl elenoic acid ester with dihydroxyphenyl-ethanol. In a similar manner to OL, OLE has shown protective effects in diseases with immunological and inflammatory profile, in which oxidative stress is a crucial mechanistic factor for their pathology, as is the case with MS [59]. OLE treatment has been found to attenuate EAE development in female C57BL/J6 mice, with myelin oligodendrocyte glycoprotein (MOG) immunization, after daily injection i.p. at 10 mg/kg daily from immunization day, with delay in disease onset and reduction in its severity up to 90%, as it was observed by the clinical scores, with decrease in MOG specific IgGs and reduction in splenocyte function similar to the control groups [60].

These effects explain the notable decrease in, the CNS tissues, infiltrating cells and the increased myelin staining, indicating low immune responses and myelin preservation of the OLE treated group, a fact that may be also derived by the reduced BBB disruption. These effects were accompanied by TNF-α decrease and IL-10 increase in spinal cord, oxidative stress modulation as seen by fluorescent dye dihydro-ethidium staining and MDA and advanced oxidation protein products decrease in spinal cord, optic nerve and cerebellum. The immunomodulation effect was also seen by the microglia modulatory potency of OLE, after LPS stimulation, decreasing the iNOS and COX-2 production, together with ROS and TNF-α diminution.

### 3.7. Ellagic Acid

**Figure 4:**
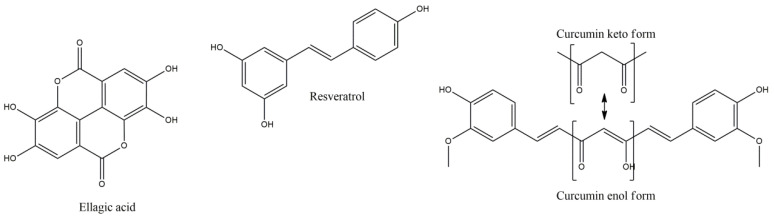
Ellagic acid, resveratrol and enol-keto form of curcumin.

Ellagic acid (EA) (Figure 4) plays a regulatory role in inflammatory processes, via suppression of important factors such as NF-kB, COX-2, IL-6 and TNF-a, via mitochondrial modulatory, iron chelating, antioxidant and axon regenerating properties [61]. In cuprizone induced demyelination increase, accompanied with locomotor and muscle tissue dysfunction, EA supplementation improved the performance of mice, via mitochondrial improvement and sirtuin-3 (Sirt-3) expression increase. Since Sirt-3 is involved in suppression of ROS production, neuronal protection and myelination, oxidative stress markers such as MDA muscle content, mitochondrial ROS levels and muscle impairment were not observed, in EA treated groups, leading to conclusion that sub-chronic EA treatment ameliorate, in a dose-dependent manner, the behavioral and muscular impairment, via mitochondrial protective and oxidation preventive mechanisms [61].

### 3.8. Resveratrol

Resveratrol is a polyphenolic stilbene compound with a wide range of activities including antioxidant, anti-inflammatory, anti-aging and neuroprotective [62]. Treatment of proteolipid protein peptide (emulsified in complete Freund’s adjuvant with mycobacterium tuberculosis) induced EAE, with resveratrol, reduces disease severity (decreasing mean and long term EAE scores), via activation of sirtuin 1 (SIRT1), preventing neuronal damage and long-term neurological dysfunction [62,63]. Reduction in leukocyte infiltration into the CNS, and induction of Th1 to Th2 anti-inflammatory cells shift, may result in these effects, together with Th17 suppression [63]. Additionally, in another study [64], high levels of apoptosis in spinal cord inflammatory cells (especially activated T cells and to a lesser extent the inactivated) was observed, in EAE-induced mice, after treatment with resveratrol, significantly down-regulating certain cytokines and chemokines, including TNF-alpha, INF-γ, IL-2, IL-9, IL-12, IL-17 and macrophage inflammatory protein-1a (MIP-1α). Resveratrol is an estrogenic modulator, and this effect was verified in this study, by activation of estrogen receptor (ER) that correlates with the aryl hydrocarbon receptor (AhR) activation and Fas and Fas ligand (FasL) expression, resulting in apoptosis of primary T cells. However, the anti-inflammatory potential of resveratrol, on EAE, is under question, since oral administration failed to prevent or alter the phenotype of inflammation in spinal cord or optic nerve [65]. Nevertheless, in this report resveratrol delayed by almost five days the onset of EAE symptoms and by two weeks the retinal ganglion cells dysfunction, with significant visual functionality.

### 3.9. Curcumin

Curcumin (CUR) (1,7-Bis [4-hydroxy-3-methoxyphenyl]-1,6-heptadiene-3,5,dione) is a natural polyphenolic phytochemical with anti-inflammatory, antioxidant, wound healing and anticancer activities, freely able to cross the BBB and regulate the CNS microenvironment, offering neuroprotective potency in various CNS diseases, with increased glutathione, HO-1, Nrf2 activation and suppressed activation of mediators such as NF-kB, COX-2, iNOS, IL-1, IL-2, IL-6 and C-reactive protein (CRP), by activated macrophages [66,67]. Furthermore, it prevents BBB disruption, induced by Th17 cells, and activates antioxidant heat shock proteins 70, sirtuins and thioredoxin [68,69]. Additionally, CUR can inhibit IL-12, and tyrosine phosphorylation of various transcription factors and their induction in T cells, regulating the production and differentiation of Th1 and Th17 cells. CUR can achieve ROS scavenging, blocking indirectly protein aggregation, with potent metal chelating capacity, mainly by its enol form, but also less potently with its methyl-catechol moiety, forming inactive complexes with Mn^2+^, Cu^2+^ and iron Fe^2+^ ions, preventing brain damage [69].

CUR has also been found to be active, in EAE Lewis rats, after oral administration of 100 and 200 mg/kg/day, from 0 until 14 days after immunization [70]. CUR decreased the clinical severity from 3.71 to 2.75 and 1.65, respectively, for each dose, with significant difference from control values. In parallel, it decreased the inflammatory cells infiltration in spinal cord and the neural Ag-specific lymphocytes proliferation, as well as the mRNA expression of cytokines IL-17, TGF-β, IL-6 and the Th17 cell proliferation and differentiation. In another study [71], dendrosome nanoparticles of CUR (PNCs, polymerized form of curcumin) were used, in order to overcome its poor bioavailability, at 12.5 mg/kg/day, starting from the day of the EAE induction or the twelfth day. PNCs reduced the development of EAE score, especially when it started from the beginning of EAE induction, and the relapse of the symptoms, causing demyelination reduction and increasing the expression of anti-inflammatory gene markers IL-10, tumor Growth Factor-b (TGF-b), IL-4 and FOXP3 regulatory transcription factor of T regulatory cells, regulating T helper cytokines production. On the other hand, pro-inflammatory gene expression such as IL-1, IL-17, TNF-a and NF-kB, was decreased, with no signal of CD8+ T helper 1 cell antibodies. These effects may partly be derived by the antioxidant potential of the PNCs that offered iNOS suppression and HO-1 and Nrf2 genes up-regulation in the spinal cord, whilst remyelinating processes may also take place, as the increase in Brain Derived Neurotropic Factor (BDNF), Nerve Growth Factor (NGF) as wells as the myelin basic protein (MBP) may dictate. In this study, there was significant increase in expression of BDNF in both EAE and PNC treated animals compared with healthy group, although the increase in PNC-treated was interestingly lower than EAE, but not significantly. Towards this direction is also the finding on mRNA levels of oligodendrocyte precursor cell markers, platelet-derived growth factor-alpha receptor (PDGF-Rα) and Olig2 (oligodendrocyte transcription factor 2), which were found up-regulated, compared to EAE control animals. However, one of the most interesting findings in this study is the preventive (when applied as pretreatment) role PNCs may play on EAE development apart from its amelioration [71].

## 4. Vitamins

### 4.1. Vitamin E

Vitamin E, conversely to total homocysteine, has been found to be decreased in serum of MS patients, whilst stress markers and cholesterol (CHL) levels were increased, with parallel decrease in the ratio of vitamin E/CHL in serum, and decreased levels of vitamin E in the demyelination plaques of MS brains, indicating possible involvement of this vitamin in MS chronic neurodegeneration [72], although, until nowadays, direct association between high levels of vitamin E and reduced risk of MS has not been found. However, in INF-β treated RRMS patients, 10 μmol/L increase in alpha-tocopherol reduced the odds of T2 magnetic resonance imaging (MRI) combined unique activity by 35.4%, whilst after two months the corresponding odds for T1 lesions were reduced by 65.4% [73,74].

**Figure 5:**
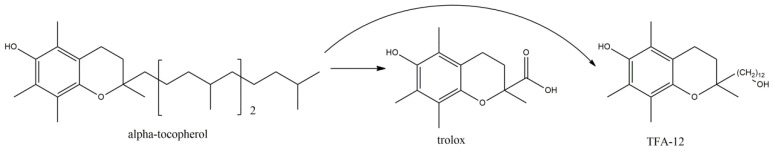
alpha-tocopherol, trolox (a widely used in medicinal chemistry synthetic derivative) and TFA-12.

Vit E (100 mg/kg) or Vit D3 (5 μg/kg) i.p. administration for up to 28 days post lesion, following local ethidium bromide (EB) injection induced demyelination, showed significant decrease in the extent of demyelination, with increased myelination intensity, after 28 days post lesion. Results were similar for both vitamins, although Vit E increased, but not significantly, the myelination intensity, compared with D3 [75]. The results depicted that vitamins shortened the recovery time two-fold compared with the control group and significantly increased (*p* < 0.001) the expression of MBP and decreased (*p* < 0.001 and *p* < 0.01, respectively, for vitamins E and D3) the levels of activated caspase-3, accentuating the remyelinating and anti-apoptotic effect of these vitamins. Furthermore, vitamin E supplementation in EB demyelinating model was accompanied by significant modulation of the acetylcholinesterase activity in various brain regions (striatum, hippocampus, cerebral cortex) and erythrocyte, accentuating its implication in cholinergic transmission. Furthermore, its protective role against demyelination was confirmed [76]. Perhaps, these effects may also derive from a modulatory activity of vitamin E on transcription factors, such as NF-kB [77], since such factors are induced throughout the disease period and their down-regulation has been confirmed with attenuation of EAE clinical symptoms. However, even though vitamin E seems to permeate the BBB it seems that it reaches in lower concentrations than blood [77]. In view of vitamin’s E encouraging results, another synthetic derivative of α-tocopherol, TFA-12 (Figure 5), belonging to tocopherol long-chain fatty alcohols, have been tested (given intraperitoneally daily in 0.39 mg/kg body weight) in 200 μg of synthetic myelin oligodendrocyte glycoprotein (MOG) induced EAE in mice [78].

TFA-12 significantly down-regulated astroglial and microglial activation in vitro, inhibiting the expression of IL-1β and TNF-α, and the mean clinical scores, in mice, was lower than 1.5, in comparison with the vehicle treated mice, which reached up to 2.5, from day twenty post induction. The anti-inflammatory effect was also confirmed by the 2.8-fold decrease in leucocytes in TFA-12-injected EAE mice, compared with controls, and the reduction in the reactive astrogliosis after TFA-12 administration, whilst the accumulation of myelin was diminished. This may partly explain the preventive effect of TFA-12 on demyelination, an effect that may also derive from the oligodendrocytes differentiation induction, which also may account for the remyelinating effects of the compound on the focal demyelinated lesions.

### 4.2. Vitamin A and Carotenoids

Vitamin A (Vit A) is a group of organic compounds as retinol, retinal, retinoic acid and structurally similar to carotenoids, with multiple actions, including hydrogen atom and electron donating effects, resulting in antioxidant, but also under certain circumstances, in pro-oxidant activity via the yielding of reactive hydroxyl radical [79]. Furthermore, indirect antioxidant effects may occur, via stimulation of peroxisome proliferator-activated receptors (PPARs) and peroxisome proliferator response elements, enhancing antioxidant enzymes expression or decrease the transcription of other inflammatory factors, such as NF-kB signal activators and transducers of transcription STAT-1 and AP-1 signaling [80]. Vit A administration (as retinyl palmitate at 25,000 IU/day) to RRMS patients, at a placebo-controlled trial, showed decreased peripheral mononuclear cell proliferation in the presence of MOG and fetal calf serum (FCS) in the culture medium, between patients receiving vitamin A and the placebo [81]. These results are in parallel with another double-blind randomized clinical trial carried out on RRMS patients, that after one year of retinyl palmitate administration with 25,000 IU/day, for 6 months, followed by 10,000 IU/day for another six months, gave significant improvement of multiple sclerosis functional composite (MSFC) in the group supplemented with vitamin A [82]. However, in the latter trial, the results in the annualized relapse rate, the differences in EDSS changes, or the volume of T2 hyper-intense lesions were not encouragingly different from the placebo groups, demonstrating that vitamin A may affect mostly the neurodegenerative path, rather than the inflammatory part of MS, but also accentuating the need for further research.

Similarly, retinoic acid (RA) enhanced the induction of Treg cells and inhibited the differentiation of Th1 and Th17 cells, whereas it promoted effector Th1 and Th17 cells and suppressed the IL-17 production, in vivo, as well as the ability of CD4+ T cells for EAE induction [83]. It also increased the secretion of IL-10 by MS B cells, without affecting TNF-alpha, in a similar to interferon-β manner [84]. These effects were retained during glatiramer and interferon-β treatment in RRMS, preserving the production of anti-inflammatory IL-10, although IFN-β alone has been shown to decrease both TNF-alpha and IL-10 production. RA also inhibited STAT3 phosphorylation that may partly be derived from reduction in the expression of IL-1β and IL-23. Thus, vitamin A may promote the differentiation of T cells, towards the immunosuppressant Th2 and Treg phenotypes, regulating relevant transcription factors, by inhibiting the generation of cytokines, such as IL-12, TNF-alpha and NO, and resulting in EAE suppression and proliferation of immune reactive cells, whilst its deficiency may account for MS pathogenesis risk [85]. RA synthesis has been shown to be important for BBB homeostasis and astrocytic control. Retinaldehyde dehydrogenase 2 (PARLDH2) is a key enzyme of RA synthesis, highly expressed in reactive astrocytes at white matter lesions, offering protective role in inflammation induced brain barriers loss, inhibiting monocytes adhesion and offering antioxidant potential, via NF-kB transcription enhancement [86]. These results render RA an endogenous protective and anti-inflammatory molecule for the neurons. Thus, it is suggested that vitamin A could be beneficial in the relief of inflammation in degenerative phase, as a complementary therapeutic support. A belief that may be accompanied by careful consideration of the dose, route and composition of vitamin A administered preparations, since modifiable plasma levels may account for the deduction of inconclusive results.

One of the categories of carotenoids is the apocarotenoids, derived from oxidative cleavage of carotenoids. Bixin belongs to this category; however, extracted at the *cis* formation, is highly unstable, and during isolation process can be isomerized into *trans*-bixin, possessing antioxidant, anti-apoptotic and anti-inflammatory properties [87]. The bixin activity on MOG peptide, induced EAE, in combination with Freund’s adjuvant and Mycobacterium tuberculosis H37Ra strain, was tested, administered at the twelfth day after immunization, intragastrically, and for eighteen consecutive days. Bixin preserved the weight loss, induced by EAE, significantly (*p* < 0.01), and the symptoms (*p* < 0.01) at 100 and 200 mg/kg in a similar manner, showing only tail paralysis [88]. The inflammation score and the infiltration of cells was reversed almost two-fold, with the expression of CD3+ cells, IL-6, 8, 10 and TNF-alpha, approaching that of control mice, and with the demyelination score to be half of the non-treated animals. Additionally, Th1 and 17 levels in spleen and CNS were significantly reduced together with the Th1 produced cytokines IFN-γ and IL-17 from Th17, respectively. The anti-inflammatory profile of bixin was completed by the observed suppression of TXNIP/NLRP3 inflammasome, with parallel decrease in IL-1β, IL-18 and caspase-1, whilst the dihydroethidium decreased staining of brain and spinal cord. The decrease in MDA and 3-nitrotyrosine, and the increase in SOD, NQO-1 and catalase, seem to improve the antioxidant capacity, potentially partly derived from Nrf2 signaling that seems to be reversed, via the intraperitoneal administration of ML385 Nrf2 inhibitor, 1 h before bixin treatment.

**Figure 6:**
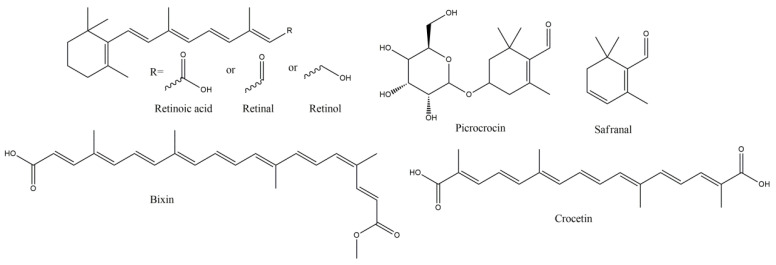
Vitamin A and Carotenoids with anti-MS potency.

Another carotenoid with cardiovascular, neural and gastrointestinal protective, cell antiproliferative and anti-inflammatory effects is crocin, and its effects are mainly attributed to its antioxidant capacity, decreasing oxidative markers, such as MDA, and giving very significant decrease in clinical impairment scores (*p* < 0.001), in EAE induced animals. Crocin is formed by the lipophilic dicarboxylic acid crocetin, di-esterified with disaccharide gentiobiose, and together with crocin 2 comprise some of the major active components of saffron, the ethanolic extract of which decreases LP and increases antioxidant levels, dose-dependently [89]. Furthermore, the ethanolic extract seems to ameliorate the spatial learning and memory deficits at MS, inducted, via intrahippocampal ethidium bromide injection, in Wistar rats, as assessed using Morris Water Maze [90]. Furthermore, total antioxidant capacity, LP products and antioxidant enzymes levels, were significantly improved, restoring the antioxidant status almost to the normal levels. The effects of crocin have been tested also at MS patients, in a double-blind, randomized and placebo-controlled trial [91]. The treated group had remarkable decrease in MDA levels (91.31 nmol/mL), in comparison to placebo (106.06 nmol/mL) (*p* = 0.01), with further improvement of TAC (*p* = 0.04), total thiol group level (*p* < 0.05), IL-17 (*p* = 0.00), TNF-a (*p* = 0.00) and DNA damage improvement (*p* = 0.00). Some other main constituents of saffron (crocus sativus) are safranal (SF) and its precursor, monoterpene glycoside, picrocrocin (Figure 6), that give the final SF under enzymatic hydrolysis by glycosidase and dehydration. SF has several neuropharmacological activities, including antioxidant, anxiolytic, anti-inflammatory and anticonvulsant, together with immunomodulatory effects [92]. SF seems to offer neuroprotection and oligodendrocytes viability increase, after glutamic acid (GA) or quinolinic acid (QA) treatment, with parallel significant (*p* < 0.001) decrease in ROS and MDA levels, with the change being dose dependent up to 10 μΜ and remain almost stable for concentrations up to 200 μΜ [93].

## 5. Polyunsaturated Fatty Acids

Omega-3 polyunsaturated fatty acids (ω-3 PUFAs) offer immunomodulatory and antioxidant activities, via decrease in inflammatory mediators and adhesion molecules, and also by giving rise to anti-inflammatory agents, such as protectins and resolvins. They significantly reduce ROS, under hydrogen peroxide stimulation, directly by autoxidation, or indirectly by regulating the expression of antioxidant enzymes as heme oxygenase-1, thioredoxin reductase-1 and SOD-Mn and up-regulating of NRF2-mediated antioxidant response [94]. Their effect on patients with RRMS, receiving 4 g/day fish oil, containing 800 mg EPA (eicosapentaenoic acid) and 1600 mg DHA (docosahexaenoic acid), per day orally, decreased TNF-α, IL-1β and 6, together with decrease in NO metabolites [95]. Consumption of EPA and DHA acids results in their increased concentration on the phospholipids and their incorporation at the expense of arachidonic acid derived eicosanoids, decreasing the production of prostaglandin E2 (PGE2), thromboxanes and various leukotrienes by the inflammatory cells, and production of eicosanoids with reduced inflammatory potency or resolvins with anti-inflammatory action [96]. Omega-3 PUFAs may decrease the production of IL-1 β and TNF-α by monocytes, and modulate the MMP-9 protein and activity levels, on peripheral blood mononuclear cells (PBMC) migration into the CNS and the BBB, causing less damage and disruption, respectively [97].

Punicic acid (PU) is a polyunsaturated omega-5 fatty acid, with a *trans* double bond conformation, at the eleventh carbon atom, and possesses high antioxidant and anti-MS activity, as it is shown by the beneficial effects of pomegranate seed oil (PSO), mainly comprising PU [98,99]. PSO, especially in nanodroplet formulation (Nano-PSO), showed significant beneficial effects, in EAE induced C57Bl naïve female mice, in much lower concentration (10% dose of the lowest active concentration of PSO) of the oil, reducing disease burden and preventing the disease onset, administered from the first day of EAE induction [98]. Additionally, nano-PSO treated groups showed significant demyelination and LP decrease, with MDA levels reaching the levels of the naïve brains following Nano-PSO treatment group. Since MS substantially affects cognitive impairment, the effect of nano-PSO was tested in placebo controlled clinical trial, where one group was given nano-PSO for three months and placebo for another three, whereas the other group was receiving placebo at first and nano-PSO the second three months [100]. The adverse effects of the nano-PSO were minimized, with significant increase in z-score of the California verbal learning test-II up to 50% [100]. Although the results in all the tests were not so profound, all the tested cognitive functions had an overall improvement, during the nano-PSO treatment trimester, in comparison to placebo group, with the results remaining high, even the following, with placebo treated, trimester, suggesting a long-lasting effect of the PSO.

Plasmalogens (PLGs) are phosphorylcholines or phosphorylethanolamines with two hydrophobic chains attached to glycerol [101]. The sn-1 chain is a nonhydrolyzable (vinyl)-ether-linked (with saturated or monounsaturated fatty alcohols) and the sn-2 is linked by ester formation with ω-3 or ω-6 polyunsaturated fatty acids [102]. Myelin is unusually rich in plasmalogen phospholipids, and plasmalogen deficient cells seem to be more susceptible to oxidative stress, whilst PLGs seem to halt the LP, although pro-oxidant capability of the oxidized PLG structure still remains (however, the radical species of vinyl ether bond oxidation are more chemically stable and less reactive). Myelin in sciatic nerve from plasmalogen-deficient (Pex7 knockout) mice was significantly more vulnerable to cooper (Cu), ortho-phenanthroline (OP) and hydrogen peroxide, mediated ROS-induced myelin compaction [103]. This effect may relate to the more efficient uptake of Cu and OP complex, whilst the antioxidant potential of PLGs may rely on the preferential oxidation of vinyl ether bond, and the adjacent PUFA of the PLGs, by radicals, rendering them more vulnerable. Furthermore, PLGs mostly interfere with propagation and not initiation of LP, since vinyl ether bond seems to interact with the lipid peroxyl radicals. Due to sterical segregation of the PUFA pool, vinyl ether is less able to approach them and offer radical reactions propagation [104]. Thus, it is also possible that LP is involved in intermodal myelin compaction and the PLGs results may directly support the role of plasmalogens as endogenous antioxidant structures for protection of ROS-vulnerable myelin.

Co-treatment with PUFA and various other antioxidant or non-antioxidant compounds for the manipulation of MS has been tested in double blind, placebo-controlled clinical study [105]. In this work, the potentiality of an ω-3 and ω-6 PUFA combination with monounsaturated fatty acids and vitamin A, α-tocopherol and high levels of γ-tocopherol (760 mg) was conducted in patients with RRMS. 64% relative decrease in relapse rate was recorded, reaching up to 72% the patients that are not receiving monoclonal antibody therapy. The cumulative probability for disability progression time had almost six-fold increase in the placebo group and even higher in the non-natalizumab treated patients, whilst no adverse effects were recorded. This effect shows that the combination of antioxidant compounds and PUFAs results in substantial improvement, since the administration of the antioxidant vitamin γ-tocopherol alone gave similar to the placebo effects. The effects of this combined supplementation were confirmed recently [106], on gait and functional capacity parameters in patients with RRMS, in a relative clinical trial, after twelve or twenty-four months of supplementation. Functional capacity was examined using various functional tests such as using six-minute walk, two sit-to-stand, timed up and go, isometric handgrip strength and leg strength functional tests and spatiotemporal gait parameters (SGP). Supplementation led to significant improvement of SGP with parallel improvement of gait deviation index (+4% in the treated compared to −10% in the placebo group). As for functional capacity, there has been a tendency of improvement in all the tests, with significant improvement at the total number of sit-to-stand cycles within sixty seconds [106]. However, the results are not always conclusive in favor of PUFAs against MS, and they do not always offer significant results in combination with MS treatments, pointing out significant limitations in the clinical studies and indicating the need for further studies [107].

**Figure 7:**
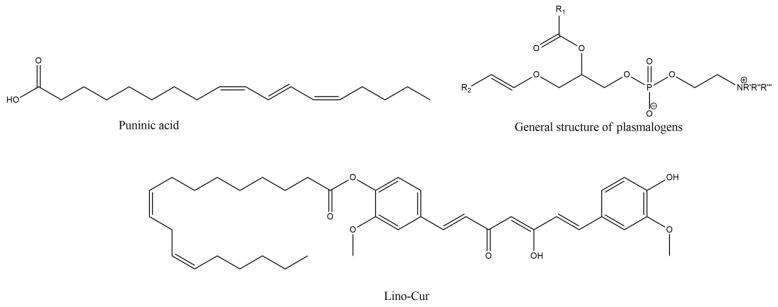
Punicic acid, general structure of plasmalogens (R’ = R’’ = R’’’ = -CH_3_ or -H, R_1_ = ω-3 or ω-6 lipid moiety, R_2_ = saturated or mono-unsaturated lipid moiety) and linoleic acid with curcumin synthetic derivative Lino-Cur.

In a recent article [108], a synthetic derivative of curcumin (CUR) and linoleic acid (Lino) was synthesized, in order to test the activity of the combined structure, in comparison to curcumin alone, on spatial memory and oxidative stress, induced by intracerebroventricular administration of ethidium bromide. The synthesis of the derivative (Lino-CUR) (Figure 7) was performed from Lino and Cur, in the presence of dimethylaminopyridine and 1-ethyl-3-(3-dimethylaminopropyl carbodiimide), at equimolar quantities, in acetone, overnight, and purification of resulted crude material with flash column silica chromatography. The distance the rodents travelled, at Morris Water Maze Test, was significantly reduced in the Lino-CUR treated group, in a dose-dependent manner, even between the Cur and the Lino-Cur groups, treated with almost the same moles of Cur, on days three and four of the administration. The most significant effect of Lino-CUR was observed on the total antioxidant capacity, which was increased in the Lino-CUR treated rats, compared with the decrease in the CUR groups, although both groups showed significant decrease in the MDA levels. The results were significantly different in favor of Lino-CUR compared with CUR group, and this decrease in LP, and oxidative stress in general, may depict the low catalase and SOD activities that were found in all the treated groups, compared to the EB group.

Another category of endogenous bioactive lipids is the aliamides (autacoid local injury antagonist amide), a group of naturally occurring N-acyl ethanolamides as palmitoyl ethanolamide (PEA), oleoyl ethanolamide (OEA) and stearoyl ethanolamide (SEA), as well as other molecules such as eicosatrienoyl ethanolamide [109]. Their main target is mast cells (MCs) and microglia, and are produced enzymatically from membrane precursors after noxious stimuli [110]. Among them, PEA bear anti-inflammatory and immune modulatory activities, with low toxicity, offering potential application against neuronal damage and pain, since its concentration is found increased in the brain and spinal cord [110]. PEA administered intraperitoneally, in a single dose, offered reduction on the clinical neurobehavioral impairment, and decrease in demyelination and axonal damage, and in inflammatory cytokine expression as well [111]. In a same manner, as previously described [43], PEA combined with LUT offers clinical signs and inflammatory genes expression improvement, accentuating also the synergistic effect aliamides may have.

## 6. Sulfur Containing Antioxidants

### 6.1. N-Acetylcysteine

**Figure 8:**
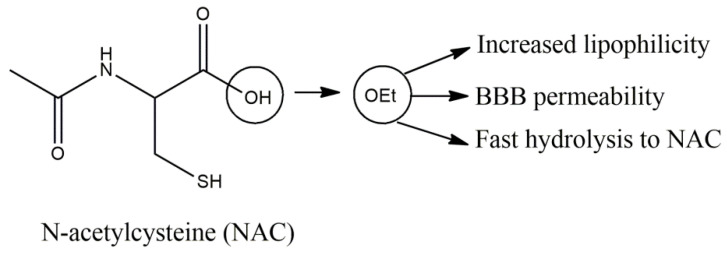
N-acetylcysteine and the properties of its ethyl ester.

N-acetylcysteine (NAC) is a very well-established antioxidant, GSH precursor, increasing GSH in brain [112]. NAC in EAE rodent models attenuated cytokine release, like TNF, the oxidative burden, by ROS scavenging, and improved clinical symptoms [113]. NAC, unlike cysteine, does not require active transport mechanisms for cellular permeation, and after cell entrance it is hydrolyzed to cysteine, which mainly assists to GSH synthesis by glutamylcysteine and glutathione synthetases. Thus, NAC is a direct and indirect antioxidant and its lipophilicity plays a vital role for its action, as is the case of the ethyl ester of NAC (Figure 8) that rapidly increases the GSH tissue content [114]. However, BBB crossing ability of NAC is disputed, and is believed that active transport after N-deacetylation is the sole crossing pathway, although in BBB inflammatory conditions, such as lipopolysaccharide (LPS) intraperitoneal administration, increased concentration of ^14^C-NAC was observed [115].

Neurovascular coupling assists the neurons to have sufficient blood flow. In various neurological disorders there has been a decrease in cerebral blood flow (CBF), either due to direct disease derived effects on vasculature, or by neuronal dysfunction, and such decreased perfusion has been seen in areas of MS patients [116]. Oxidative stress is capable of altering CBF within the brain and decreased perfusion has been correlated with higher functional disability [117]. NAC inhibits serum kinases affecting redox-sensitive mechanisms, and regulating platelet aggregating enzymes, improving vascular smooth muscle cells performance [114]. NAC, intravenously, once per week, in MS patients, with PP and RRMS, showed significantly increased cerebral glucose metabolism in caudate, inferior frontal gyrus, lateral temporal gyrus, and middle temporal gyrus, tested by integrated Positron Emission Tomography, using 18F-fluorodeoxyglucose, and significant improvement also at the self-reported results concerning cognition and attention [118].

### 6.2. S-allyl-L-cysteine

S-allyl-L-cysteine (SAC) is a water soluble organosulfur, garlic derived, compound, with antioxidant and various other actions. It offers survival and axonal branching points increase, with learning and cognitive deficits and memory consolidation improvement, via oxidative stress and neuroinflammation suppressing ability [119,120]. When endoplasmic reticulum (ER) fails to manipulate the load of misfolded, or unfolded proteins, it may result in ER stress and apoptosis induction, with stress related factors, such as caspase-12, activation taking place in this direction, via calpain protease activity. SAC can inhibit calpain induced cell death and offer neuroprotection, as well as its hydrophobic S-methyl, S-ethyl and S-propyl garlic derivatives that also offer neuroprotection [119].

In MS treatment, SAC per os administration, at 50 mg/kg/day, in MOG induced female mice, showed clinical signs and severity alleviation, with TNF-alpha, IL-17 and MMP-9 reduction, and activity-dependent neuroprotector homeobox (ADNP) and microtubule-associated proteins 1A/1 B increase [121]. Additionally, attenuation of immune cell infiltration, demyelination and axonal loss, in spinal cord, was succeeded. These results are in accordance with Escibano et al. showing that SAC could reverse the oxidative and nitrosative damages, induced by MOG, in spinal cord and the brain, improving the glutathione levels and attenuating mitochondrial damage [122]. The beneficial effects of SAC were better than DMF, and the oxidative markers decrease was positively correlated with the established clinical scores, depicting that neuroprotective efficiency of SAC goes in parallel with its antioxidant capacity.

### 6.3. H-1,2-dithiole-3-thione

**Figure 9:**
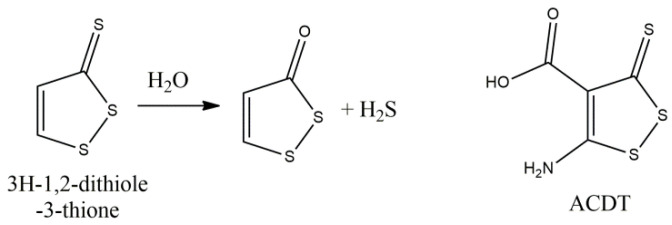
Hydrogen sulfide donating effect of 3H-1,2-ditione-3-thione and its derivative ACDT.

3H-1,2-dithiole-3-thione (DTN) (Figure 9) is the simplest derivative of the sulfur containing dithiolethiones, with antioxidant and gene inducing capacity, mainly derived from activation of Nrf2, via Keap1 eradication, and nuclear localization of the transcription factor, giving rise to detoxifying enzymes [123]. Dithiolethiones, as well as dithiolones, offer hydrogen sulfide formation in a spontaneous, via hydrolysis, way, and indirectly after metabolic transformation and dithiolane ring opening with subsequent hydrolysis [124]. DTN possesses anti-inflammatory characteristics and offers beneficial effects, in EAE, with low disease scores (almost 65% compared to vehicle) that were reversed after treatment withdrawal [125]. DTN was able to reduce Th1 and Th17 cells infiltration in CNS, as well as their peripheral differentiation, as it was shown by the analysis of CD4+ T cells and of CD4+ IFNγ- and IL-17-expressing T cells, in the brain and spinals cord, and in the latter case, in splenocytes. Additionally, it suppressed the LPS induced expression of cytokines and stimulatory molecules, by dendritic cells (DCs), with a dramatic decrease in IL-1β and DCs maturation. This effect may be related to the Nrf2 activation by DTN, leading to an increase in HO-1 and phase II antioxidant enzymes NQO1, GCLC, followed by decrease in IL-23, iNOS, GM-CSF (Granulocyte macrophage colony-stimulating factor, indicative of DCs development and maturation) and microglia activation, affecting both the peripheral and the CNS immune cells. Similar results were accomplished by a synthetic derivative of DTN, 3H-(1,2)dithiole-4-carboxylic acid ethyl ester (ACDT), by the same team [126]. ACDT delayed disease onset and severity, and administered during remission phases, effectively decreased the relapse phase in RR EAE, by prevention of T cells infiltration, in CNS, and suppression of microglial cells, exhibiting anti-inflammatory properties, as the reduction in mediators such as TNF-alpha, myeloperoxidase and CD40 suggests. The effect of ACDT on matrix metalloproteinases 3 and 9 led to BBB integrity and disruption lessening, a finding that may be related also to the mediation of astrocytes generation.

### 6.4. A-Lipoic Acid

a-Lipoic acid (LA) is an antioxidant and anti-inflammatory molecule that as such, or in its reduced form, dihydrolipoate (DHLA), offers reactive oxygen and nitrogen species scavenging, especially DHLA, and transitional metal chelating ability, such as labile iron, diminishing the oxidative burden of the tissues and reactivating-reducing vital antioxidant molecules, such as alpha-tocopherol, vitamin C and glutathione [127]. Furthermore, LA offers anti-inflammatory effects, via regulation of transcription factors, such as NF-kB, and is a promising therapy for MS treatment, confronting the accumulating disabilities of MS [128]. This effect was clinically shown in a placebo-controlled pilot trial of progressive MS patients [129]. Significant decrease in percent change in brain volume (PCBV) of LA treated patients and with fewer falls and brain atrophy were documented. Furthermore, LA stimulates cyclic adenosine monophosphate (cAMP), in mononuclear cells, resulting in a less inflammatory phenotype in MS cells and monocyte derived macrophages, with inhibitory effect on the secretion of cytokines and phagocytosis [130]. LA suppressed the number of Th1 and Th17 cells and reduced their infiltration into CNS (spinal cord and cerebellum), with PPAR-γ induction, centrally and peripherally, and T-reg cells enhancement, offering modulation of adaptive immunity and further antioxidant mechanistic effects [131]. Furthermore, inflammatory mediators’ modulation, such as MMP-9, soluble adhesion molecule (ICAM), IFN-γ and TGF-β, has been reported in animals and humans [131,132]. The LA administration at the early or late suppression phase (8 days and 13 post MOG immunization, respectively), showed 87% and 66% reduction, in the optic nerve and axonal damage, and significant reduction in CD4+ and CD11b+ cells [133]. These effects showed that, both early and late administration of LA may abolish EAE impairment, but with a more robust way at the first case, whilst subcutaneous treatment, seams more effective, than per os, if we take into account the fluctuated bioavailability of LA [127,133].

### 6.5. Biotin

Biotin (vitamin B7 or vitamin H) is involved in utilization of vital structural molecules of the tissues (lipids, sugars, proteins), and its supplementation may account for decrease in LP and improved mitochondrial function, improving their radical formation potential [134]. High doses of biotin supplementation (median = 300 mg/day, divided in three doses, almost 10,000 times the adequate daily intake) had positive results in more than 90% of patients with SPMS and PPMS, and the clinical effects were obvious after two or even eight months after the onset of treatment [135]. Biotin activates enzymes concerned with energy and myelin production, with positive effects on chronic optic neuropathy and myelopathy. In another randomized, double-blind, placebo-controlled study, 100 mg biotin, thrice daily for 24 months, led to statistically significant number of patients that could achieve the primary endpoint of EDSS decrease, more than or equal to 1 point or 20% decrease in 25-foot walk time. An improved clinical change was achieved, and the safety profile remained similar to that of the placebo group [136]. However, biotin should not be considered harmless, since transient myopathy and association of new disease activity, via its supplementation, has occurred that, combined with the relevant lack of affectivity in recent trials, renders it not recommendable for patients with MS [137].

### 6.6. Thiamine

Thiamine (vitamin B1) seems to possess antioxidant potency, via formal hydrogen transfer (FHT) and radical adduct formation mechanisms, and has been shown to inhibit LP in rat liver microsomes, as well as of oleic acid in vitro [138]. Thiamine has shown to abrogate the fatigue, related to dysfunction of the mechanisms of intracellular transport (oxidative phosphorylation in mitochondria) or enzymatic abnormalities, in MS patients [139]. Thiamine deficiency (TD) is associated with C-C Chemokine Ligand 2 (CCL2) induction, in neurons involved in the development of EAE, in MOG induced mice [140]. TD deteriorated the clinical scores and the spinal cord pathological alterations, via microglial activation, leucocyte infiltration and Th1 and Th17 cells increase. Furthermore, TD mice showed peripheral proliferation in the lymph node and spleen and migration of T lymphocytes, posing the issue of normal thiamine levels as an important factor for the modulation of the disease.

### 6.7. Sulfarophane and Moringin

**Figure 10:**
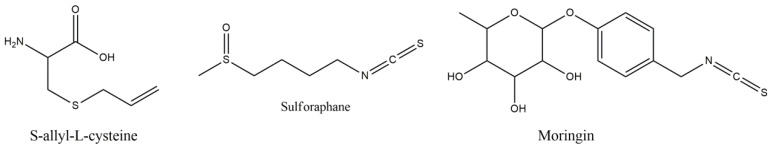
Structures of S-allyl-L-cysteine, Sulforaphane and Moringin.

Among the sulfur containing bioactive molecules, sulfarophane (SFN) (Figure 10) is a naturally occurring isothiocyanate, with cytoprotective and chemopreventive effects, via indirect antioxidant activity by NRF2 activation that subsequently leads to thioredoxine reductase-1, heme oxygenase-1, glutathione reductase and quinone oxireductase-1 expression induction [141]. However, these effects are highly related to the concentration of SFN that at low doses seems to result in up-regulation of antioxidant enzymes and at higher doses lead to cytotoxic effects, via apoptosis and DNA damaging responses [142]. The effect of SFN on MS has been tested in EAE model of mice, induced with subcutaneous myelin oligodendrocyte glycoprotein peptide [143]. Intraperitoneal SFN administration, of 50 mg/kg, inhibited the inflammatory processes, induced by T cells, and increased the anti-inflammatory IL-10 production, with parallel BBB protection and matrix metalloproteinase 9 reduction. SFN improved the behavioral deficits and reduce the demyelination and inflammatory cells infiltration in the spinal cord, with reduction in NO levels and an increase in GSH and NQO-1 activity [144]. Moringin (MR, 4-[α-L-rhamnopyranosyloxy]-benzyl isothiocyanate), is another isothiocyanate O-glycosylated phenol derivative, that offers antioxidant and anti-inflammatory, neuroprotective effects in pre-treated hydrogen peroxide induced apoptosis [145]. Moringin administration, 1 week before and 4 weeks after EAE induction, showed Wnt–β-catenin pathway (Wnt–β-catenin-dependent pathway consists of secreted signaling proteins which activate β-catenin) normalization, inhibition of GSK3β enzyme, responsible for phosphorylation of β-catenin, and regulation of T cell activation, with inflammatory mediators’ suppression, by PPARγ activation [146]. These effects also led to caspase-9 cleavage and apoptosis attenuation, via parallel increase in Nrf2 expression.

## 7. Conclusions

**Figure 11:**
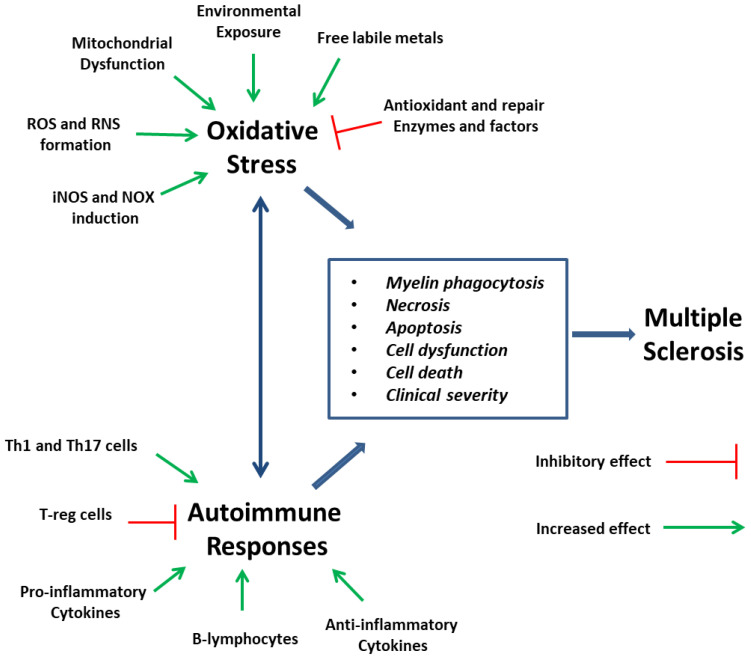
Potential levels of implication of natural antioxidants in the MS initiation and progression.

In this review, we tried to analyze the role of naturally derived or combined derivatives of natural compounds, with well-known antioxidant potential, in MS progression modulation, at the level of clinical progression, demyelination, remyelination, immune infiltration reduction and various other pathobiological mechanisms that are induced during the MS progression (Figure 11). In most of them, evidence suggests that they can have considerable role in MS pathogenesis, decelerating its progression or improving some of its unfavorable clinical characteristics, such as fatigue, cognitive dysfunction and paralysis, and their deficiency may account or relate to MS deterioration. Furthermore, many oxidative and inflammatory markers have shown to be substantially reduced, as the levels of their gene expression or their activity shows, offering advanced protection towards two main directions that affect the MS progression. Thus, the research towards the antioxidant compounds, with multi-targeting and disease modulating potential, reveal that more trials are needed for the establishment of new treatment or co-treatment approaches in MS, since many of these activities may remain inconclusive and require clinical verification. However, they also shed light into many unexplored pathways of MS pathology, clarifying its etiology that may open new opportunities for efficient treatments, or the development of naturally derived antioxidant compounds, or novel lead compounds to be used for more active and competent therapeutic agents.
